# Anastomotic leakage increases the risk of major low anterior resection syndrome 3 years after rectal cancer surgery

**DOI:** 10.1111/codi.70423

**Published:** 2026-03-16

**Authors:** Anders Gerdin, Jenny Häggström, Jennifer Park, Marie‐Louise Lydrup, Peter Matthiessen, Henrik Jutesten, Sofia Sandberg, Eva Angenete, Martin Rutegård

**Affiliations:** ^1^ Department of Diagnostics and Intervention Umeå University Umeå Sweden; ^2^ Department of Statistics, Umeå School of Business, Economics and Statistics Umeå University Umeå Sweden; ^3^ SSORG–Scandinavian Surgical Outcomes Research Group, Department of Surgery, Sahlgrenska University Hospital, Sahlgrenska Academy University of Gothenburg Gothenburg Sweden; ^4^ Department of Surgery Sahlgrenska University Hospital Gothenburg Sweden; ^5^ Department of Surgery, Skåne University Hospital Lund University Malmö Sweden; ^6^ Department of Surgery, Skåne University Hospital Lund University Lund Sweden; ^7^ Department of Surgery, Faculty of Medicine and Health Örebro University Örebro Sweden

**Keywords:** anastomotic leakage, anterior resection, bowel dysfunction, low anterior resection syndrome, patient‐reported outcomes, rectal cancer

## Abstract

**Background:**

Anastomotic leakage is a serious complication following anterior resection for rectal cancer and may increase the risk of long‐term bowel dysfunction. This study aimed to assess the long‐term impact of anastomotic leakage on major low anterior resection syndrome (major LARS) at a uniform follow‐up time.

**Methods:**

We conducted a nationwide cohort study using the Swedish Colorectal Cancer Registry. Patients who underwent anterior resection for rectal cancer between 2015 and 2017 received the validated LARS questionnaire by mail 3 years after surgery. The primary outcome was major LARS among patients without a permanent stoma. Propensity score weighting was used to adjust for confounding, with covariates chosen using a directed acyclic graph. Sensitivity analyses included a dose–response analysis based on reoperation and an evaluation of a composite outcome of major LARS or permanent stoma.

**Results:**

Of 1778 patients contacted, 1178 responded (66.2%). Among 1033 stoma‐free patients, 52 (5.0%) had experienced a symptomatic anastomotic leak. Major LARS was reported in 69.2% and 52.9% of patients with and without leakage, respectively. Symptomatic anastomotic leakage increased the risk of major LARS (OR 2.09; 95% CI: 1.13–3.87) and this risk was higher in patients requiring reintervention (OR 2.78; 95% CI: 0.87–8.91) and when including permanent stoma in the outcome (OR 3.90; 95% CI: 2.20–6.91).

**Conclusion:**

Anastomotic leakage significantly increased the risk of major LARS 3 years after anterior resection for rectal cancer. These findings underscore the importance of preventing anastomotic leakage to reduce long‐term functional morbidity in patients who survive rectal cancer.

**What does this paper add to the literature?**

Previous studies indicate that anastomotic leakage impairs bowel function after rectal cancer resection, but the studies are limited by small samples and inconsistent follow‐up. This large, nationwide study with uniform 3‐year follow‐up provides strong evidence that leakage increases the risk of major low anterior resection syndrome, underscoring the importance of leak prevention and patient counselling.

## INTRODUCTION

Curative surgical treatment for cancer in the upper and mid rectum commonly consists of anterior resection [1], a sphincter‐preserving operation aiming for bowel continuity. There are numerous short‐ and long‐term consequences of anterior resection, while the most feared complication is anastomotic leakage [2]. Anastomotic leakage after anterior resection for rectal cancer is common (20%) [3, 4] and poses a significant short‐term risk, with a higher 90‐day mortality rate [5]. Regardless of leakage, there is a substantial risk of bowel dysfunction after anterior resection [6], often referred to as low anterior resection syndrome (LARS) [7], with a reported risk range of 23%–77% [6, 7, 8, 9, 10, 11]. This syndrome consists of disordered bowel function after rectal resection, leading to a detriment in quality of life [12]. A validated questionnaire and scoring system for LARS [13] is commonly used to grade symptoms such as increased stool frequency, faecal incontinence, urgency and bowel‐emptying difficulties and previous studies have suggested that anastomotic leakage worsens LARS [6, 7, 14]. However, these studies are comparatively small and, most importantly, include LARS questionnaires completed at different time points after surgery. Therefore, there is a need to conduct large population‐based studies on LARS in relation to anastomotic leakage with consistent follow‐up times.

This registry‐based study aimed to evaluate LARS after anterior resection for rectal cancer as a function of anastomotic leakage. Our main hypothesis was that postoperative symptomatic anastomotic leakage increased the prevalence of major LARS 3 years after surgery in stoma‐free patients.

## METHOD

### Study design

This was a nationwide prospective cohort study based on the Swedish Colorectal Cancer Registry (SCRCR). The SCRCR was established in 1995 and includes all hospitals that perform surgery for rectal cancer in Sweden [15]. Rectal cancer is defined in the SCRCR as a bowel adenocarcinoma with its inferior margin 15 cm from the anal verge. The registry contains demographic, clinical and tumour‐related variables, including neoadjuvant treatment, intraoperative data and information on postoperative complications as well as long‐term outcomes. The most recent nationwide validation of the SCRCR was published in 2024, assessing data registered in 2015. It reported a high level of validity for pathology and recurrence data. However, detailed reporting on postoperative complications needs improvement, particularly for mild complications [16]. Patients operated with anterior resection for rectal cancer during 2015–2017 were identified through the SCRCR. Mailed envelopes including informed consent, LARS questionnaires and study‐specific questions on stoma status were sent to patients 3 years (± 6 weeks) after anterior resection.

### Exposure

The exposure in this study was anastomotic leakage after anterior resection for rectal cancer. The SCRCR registry does not provide a formal definition of anastomotic leakage, and anastomotic leakage in the registry thus reflects the clinical assessment (documented in the medical record) of anastomotic leakage diagnosed within 30 days after the primary operation. The severity of anastomotic leakage is not categorized according to the International Study Group of Rectal Cancer [17] in the SCRCR. However, it is possible to determine whether the anastomotic leakage necessitated a reoperation. Reoperation was defined as an unplanned laparotomy or laparoscopy within 30 days of the index surgery.

### Outcome

The primary outcome was patient‐reported major LARS 3 years after anterior resection for rectal cancer. The LARS‐score was measured with the validated LARS questionnaire [13], from which the sum of all individual symptoms was calculated, providing an overall score of bowel dysfunction: No LARS (0–20 points), minor LARS (21–29 points) and major LARS (30–42 points). The secondary outcome was major LARS or the presence of a permanent stoma among all responding patients.

### Statistical analysis

**FIGURE 1:**
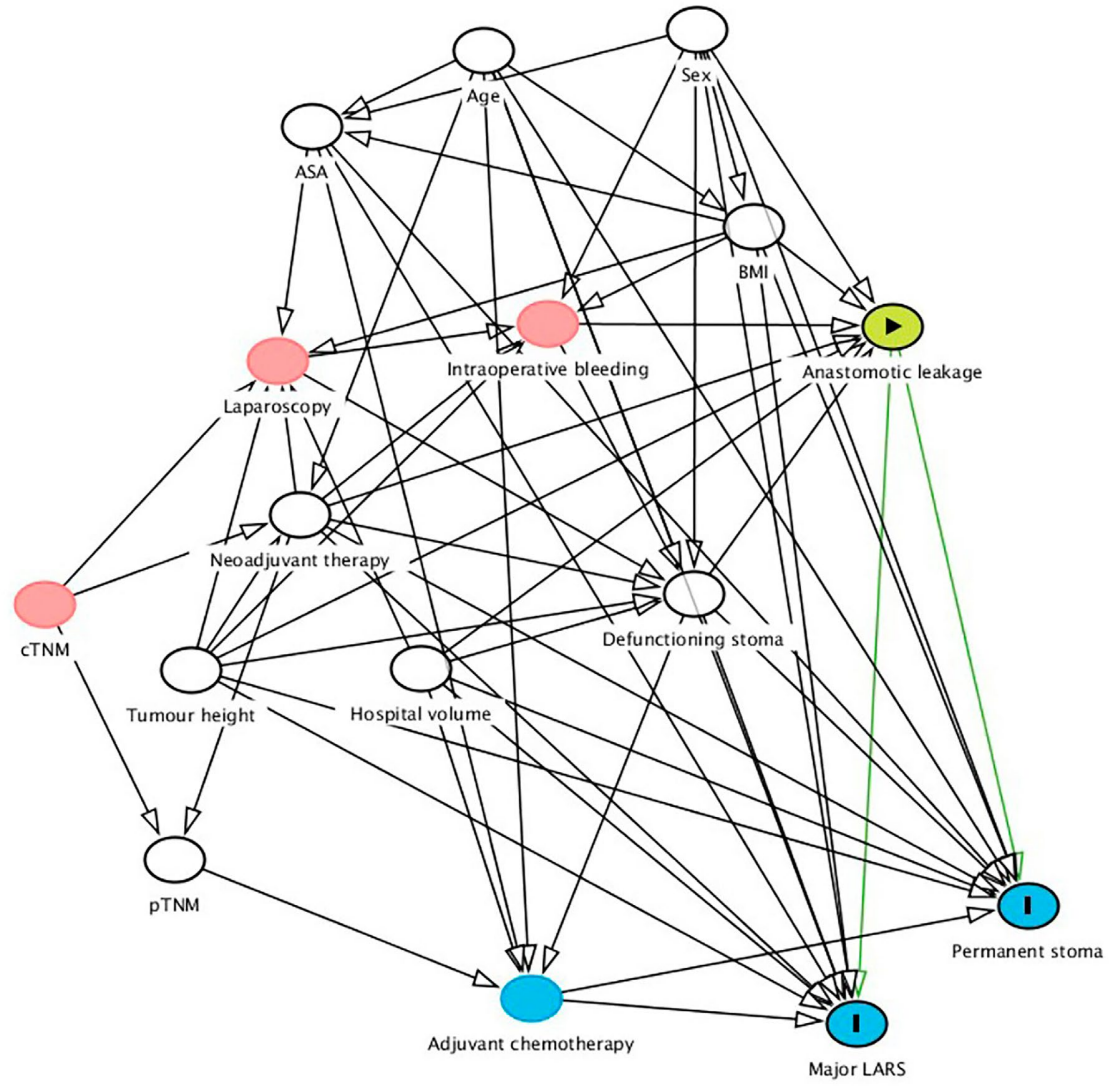
Directed acyclic graph. The green circle indicates the exposure. The blue circumscribed circle indicates outcomes. White or red circles indicate ancestors of the exposure and the outcomes, whereas white circles denote adjusted confounders sufficient to estimate the total effect. Blue uncircumscribed circles indicate mediators on the causal pathway between the exposure and the outcomes.

Baseline characteristics were assessed in relation to anastomotic leakage or no leakage. Confounders were chosen using a causal diagram [18] (Figure 1). These confounders were: ASA (American Society of Anaesthesiologists' fitness grade I, II, or III–IV; categorical), age (continuous), body mass index (BMI; continuous), defunctioning stoma (yes or no; categorical), hospital volume (continuous), neoadjuvant therapy (radiotherapy or chemoradiotherapy; yes or no; categorical), sex (male or female; categorical), tumour height (continuous) and pathological tumour stage (p/ypTNM stage; categorical). Propensity score weighting [19] was applied to adjust for potential confounding, with the aim of emulating a randomized trial in which all patients have the same likelihood of experiencing anastomotic leakage. Propensity scores were estimated using the SuperLearner algorithm [20], an ensemble machine learning technique that employs cross‐validation to select the optimal weighted combination of a set of prediction algorithms based on predictive performance. The SuperLearner incorporated logistic regression, lasso logistic regression, random forest and extreme gradient boosting as prediction algorithms. An absolute mean standardized difference of less than 0.25 was considered indicative of an acceptable balance after weighting, whereas a value below 0.10 indicated good balance [21]. The association between the exposure and outcome, in terms of the odds ratio, was estimated using a propensity score weighted logistic regression with the exposure as the only covariate. In addition, propensity score weighted outcomes were utilized to estimate the risk difference. Confidence intervals were estimated using an asymptotic variance estimator [22].

Multiple imputation by chained equations [23] was used to handle missing values, and the estimates from 10 imputed data sets were pooled according to Rubin's rules [24]. All analyses were performed using R 4.4.0 statistical software [25].

The main analysis used the exposure of any leakage in relation to the outcome major LARS. Several sensitivity analyses were conducted. Firstly, a dose–response analysis was performed, with a subdivided exposure (no leakage, leakage without reoperation and leakage with reoperation). Secondly, the composite outcome major LARS or permanent stoma was evaluated, as the latter might be a consequence of both leakage itself and major LARS.

## RESULTS

### Patient characteristics

**FIGURE 2:**
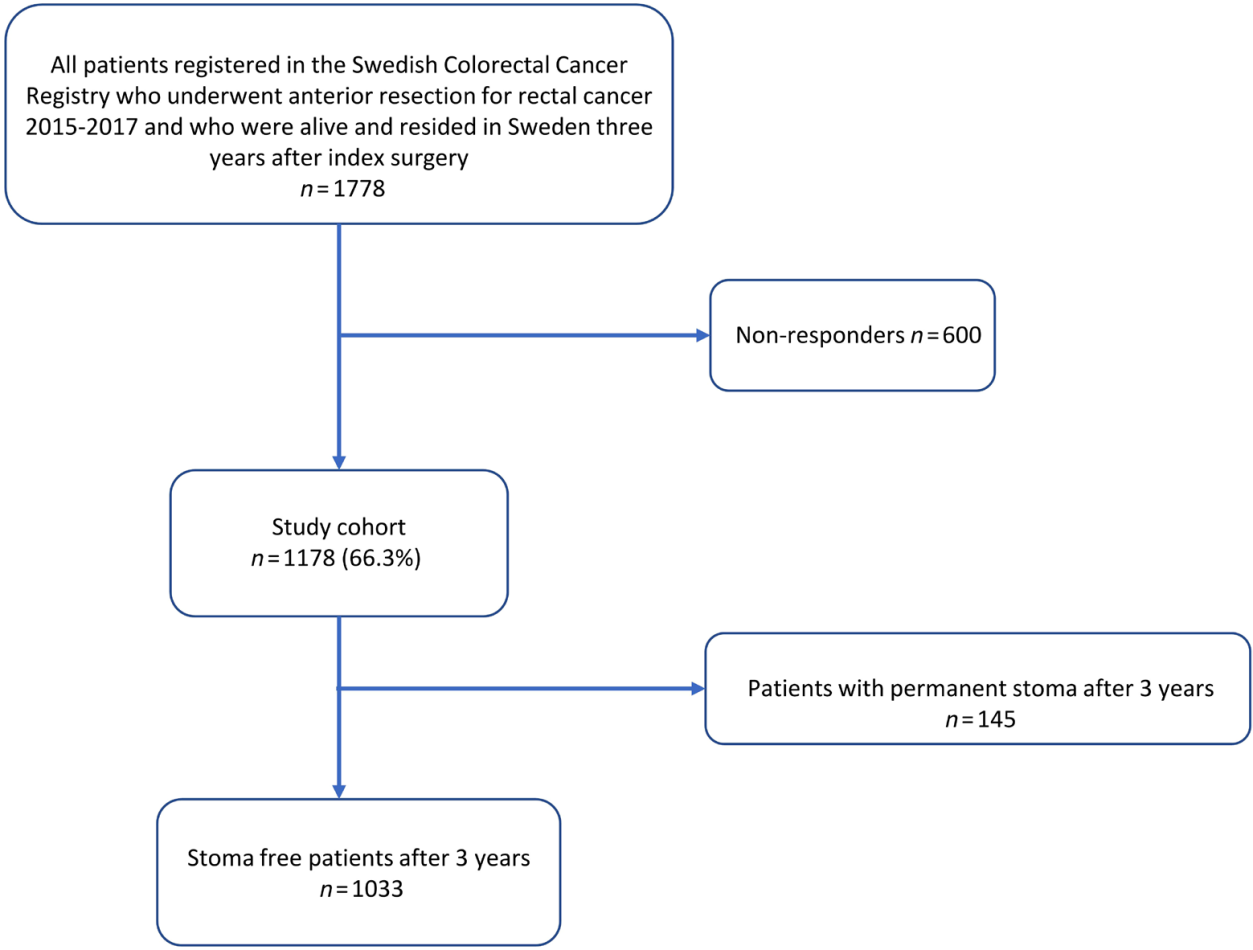
Study flowchart.

A total of 1778 patients who underwent anterior resection for rectal cancer, and were still alive and resident in Sweden, received the questionnaire. Out of these, 1178 responded, amounting to a response rate of 66.2%. Of these, 1033 patients were stoma‐free at 3 years and included in the main analysis (Figure 2). Fifty‐two (5.0%) of the latter patients had a registered anastomotic leakage. Table 1 compares the baseline characteristics of patients with and without leakage, in those without a permanent stoma at 3 years after surgery. Anastomotic leakage was more common in younger patients, men, those receiving neoadjuvant treatment, and in patients with increased intraoperative bleeding (Table 1).

**TABLE 1:** Baseline characteristics of 1033 responding patients without permanent stoma 3 years after operated with anterior resection for rectal cancer in Sweden 2015–2017, by anastomotic leakage.

	No leak (*N* = 981)	Leak (*N* = 52)	Overall (*N* = 1033)
Age			
Median (IQR)	67 (60; 72)	64 (58; 69)	67.0 (60; 72)
Missing	1 (0.1%)	0	1 (0.1%)
Sex			
Female	410 (41.8%)	16 (30.8%)	426 (41.2%)
Male	570 (58.1%)	36 (69.2%)	606 (58.7%)
Missing	1 (0.1%)	0	1 (0.1%)
Body mass index (kg/m^2^)			
Median (IQR)	25.5 (23.3; 28.1)	26.0 (23.7; 30.2)	25.5 (23.3; 28.2)
Missing	17 (1.7%)	0	17 (1.6%)
ASA			
I	262 (26.7%)	13 (25.0%)	275 (26.6%)
II	556 (56.7%)	30 (57.7%)	586 (56.7%)
III–V	151 (15.4%)	8 (15.4%)	159 (15.4%)
Missing	12 (1.2%)	1 (1.9%)	13 (1.3%)
Hospital volume (annual)			
Median (IQR)	20.3 (13.7; 26.3)	22.3 (16.0; 33.3)	20.3 (14.0; 26.3)
Tumour level (cm)			
≤12	726 (74.0%)	40 (76.9%)	766 (74.2%)
13–15	251 (25.6%)	11 (21.2%)	262 (25.4%)
Missing	4 (0.4%)	1 (1.9%)	5 (0.5%)

In the cohort of all responders (*n* = 1178), including those with a permanent stoma at follow‐up, 104 patients (8.8%) had a reported anastomotic leakage (Table S1). The overall patterns were similar to those seen in the main cohort (Table 1): leakage was more common in younger patients, men, those receiving neoadjuvant treatment, and was also related to increased perioperative bleeding.

### Non‐responders

Among the 600 non‐responders (Table S2), 66 patients (11.0% vs. 8.8% in the entire responding cohort) had a registered anastomotic leakage. Compared with responders, non‐responders had higher comorbidity (ASA III: 20.8% vs. 15.8%), more intraoperative bleeding (150 mL vs. 100 mL), and underwent minimally invasive surgery to a lesser degree (49% vs. 56%).

### Covariate balance

The achieved balance after propensity score weighting was acceptable, with all covariates showing absolute standardized differences below 0.25 in all analyses for all imputed datasets, except one covariate in the analysis comparing leakage with the need for reoperation to no leakage. Balance plots are presented in Figures S1–S4.

### Anastomotic leakage and LARS: Main analysis

Major LARS was present in 69.2% and 52.9% of patients with and without leakage, respectively. After adjustment for confounders, the OR of major LARS comparing patients with an anastomotic leakage to those without was 2.09 (95% CI: 1.13–3.87). This corresponded to an adjusted mean risk difference of 17.5% (95% CI: 4.2%–30.8%).

### Anastomotic leakage and LARS: Sensitivity analyses

In patients without a permanent stoma at follow‐up, 36 (3.5%) patients had a leakage without the need for reoperation, while 16 (1.5%) patients required reoperation for leakage. This rendered ORs for major LARS of 1.85 (95% CI: 0.90–3.83) and 2.78 (95% CI: 0.87–8.91), respectively. Among responders, permanent stoma was present in 49/101 (48.5%) patients with leakage and in 85/1068 (8.0%) patients without leakage (three and eight patients had missing data for stoma status, respectively). The risk of major LARS or permanent stoma was substantially increased in patients with leakage, compared to those without (OR 3.90; 95% CI: 2.20–6.91).

## DISCUSSION

Our study confirmed that anastomotic leakage following anterior resection for rectal cancer increased the risk of major LARS, corroborating previous findings [1, 2, 6]. This relationship was more pronounced in patients who required reoperation, possibly strengthening the causal link and highlighting the potential impact of more severe leakage. When incorporating permanent stoma prevalence into the outcome, an even stronger effect was detected.

While earlier studies such as Bregendahl et al. and Hain et al. observed similar trends, they were limited by small sample sizes or heterogeneous follow‐up intervals [8, 9]. In contrast, our population‐based design with a uniform 3‐year follow‐up strengthens the causal interpretation of this association. The increased risk of major LARS observed in patients requiring reoperation and for the composite outcome including permanent stoma supports a dose–response relationship, consistent with findings by Jutesten et al. [6]. The present study has contributed robust, population‐based evidence with uniform follow‐up and, along with previous research, highlights the long‐term consequences of anastomotic leakage.

Our findings emphasize the need for improved perioperative strategies to minimize leakage and mitigate its functional consequences. It also indicates that it is important to include the increased risk of LARS in the treatment strategy of anastomotic leakage. Future research should aim to reduce the occurrence and understand the causes of anastomotic leakage. In addition, greater attention should be directed to postoperative rehabilitation to support functional recovery in affected patients. Equally important is providing patients with clear information about the potential impact of anastomotic leakage on long‐term bowel function. For selected patients, the option of a permanent stoma needs to be considered.

The major strengths of this study include its prospective design, large sample size and the use of multiple imputation to handle missing data. Additionally, the use of a validated LARS questionnaire [13] and well‐established registry data [15] enhances the reliability of our findings. Moreover, the homogeneity of follow‐up at 3 years post‐surgery provides a clear temporal relationship between anastomotic leakage and LARS. By assessing all patients at the same postoperative interval, variations in symptom reporting due to differing follow‐up durations are minimized.

However, several limitations must be acknowledged. Potential misclassification of anastomotic leakage remains a concern [26], as the registry neither defines nor categorizes leakage and its severity based on established criteria [17]. When interpreting these findings, it is important to also acknowledge the possibility of underreporting in the SCRCR [27]. This may affect mild or asymptomatic leaks in particular, which are less likely to be detected and registered, potentially leading to underestimation of the true association. Additionally, response bias due to the 66.2% response rate could influence findings, as non‐responders may differ systematically from responders. The characteristics of non‐responders with anastomotic leakage differed in several aspects from those included in the main cohort. While some patterns were consistent, such as the association of leakage with sex, neoadjuvant therapy and bleeding, other characteristics differed, including a smaller age difference, higher ASA scores and less frequent use of diverting stomas in the non‐responding group. These findings suggest that non‐responders may constitute a clinically distinct subgroup, which could influence both the interpretation and generalisability of patient‐reported outcomes. Notably, the higher leakage rate among non‐responders compared with responders suggests that patients who experience postoperative complications may be less likely to return questionnaires, a pattern also described in previous registry‐based studies [6]. The limited data on postoperative management strategies further limit our ability to evaluate potential factors that contribute to LARS and its severity. Furthermore, information on the extent of mesorectal excision and anastomotic height is not recorded in the SCRCR, where tumour height served as the closest available proxy for these admittedly important surgical details.

In conclusion, this study indicates that anastomotic leakage significantly increases the risk of major LARS, particularly in patients requiring reoperation. These findings highlight the critical need for preventing anastomotic leakage and long‐term follow‐up to address bowel dysfunction in patients who survive rectal cancer, in particular those with a previous leakage and a preserved anastomosis.

## AUTHOR CONTRIBUTIONS

**Anders Gerdin:** Conceptualization; methodology; software; data curation; investigation; validation; formal analysis; visualization; project administration; writing—original draft; writing—review and editing. **Jenny Häggström:** Conceptualization; methodology; software; data curation; investigation; validation; formal analysis; writing—review and editing. **Jennifer Park:** Conceptualization; investigation; validation; writing—review and editing. **Marie‐Louise Lydrup:** Conceptualization; investigation; validation; writing—review and editing. **Peter Matthiessen:** Conceptualization; investigation; validation; writing—review and editing. **Henrik Jutesten:** Conceptualization; investigation; validation; writing—review and editing. **Sofia Sandberg:** Conceptualization; investigation; validation; writing – review and editing. **Eva Angenete:** Conceptualization; investigation; validation; writing – review and editing. **Martin Rutegård:** Conceptualization; methodology; software; data curation; investigation; validation; formal analysis; supervision; funding acquisition; visualization; project administration; resources; writing—review and editing.

## FUNDING INFORMATION

Swedish Cancer Society (23 3056 Fk), Region Västerbotten (RV‐991591).

## CONFLICT OF INTEREST STATEMENT

None declared.

## ETHICS APPROVAL STATEMENT

Dnr 2016/922, dnr 2017/946, dnr 2021‐00609.

## PERMISSION TO REPRODUCE MATERIAL FROM OTHER SOURCES

No material from other sources was included in this article.

## Supporting information


**Supplementary Table 1.** Baseline characteristics of 1178 responding patients operated with anterior resection for rectal cancer in Sweden, 2015–2017, by anastomotic leakage.
**Supplementary Table 2**. Baseline characteristics of 600 non‐responding patients operated with anterior resection for rectal cancer in Sweden, 2015–2017, by anastomotic leakage.
**Supplementary Figure 1**. Covariate balance across 10 imputed datasets, for responding patients without permanent stoma. Exposure = leakage versus no leakage.
**Supplementary Figure 2**. Covariate balance across 10 imputed datasets, for responding patients. Exposure = leakage without reintervention versus no leakage.
**Supplementary Figure 3**. Covariate balance across 10 imputed datasets, for responding patients. Exposure = leakage with reintervention versus no leakage.
**Supplementary Figure 4**. Covariate balance across 10 imputed datasets, for all patients. Exposure = leakage vs. no leakage.

## Data Availability

The data that support the findings of this study can be provided if approved upon request from the Swedish colorectal cancer registry (https://scrcr.se/).
