# Supplementary material for: Anastomotic leakage increases the risk of major low anterior resection syndrome 3 years after rectal cancer surgery
Source: Colorectal Dis. 2026 Mar 16;28(3):e70423. doi: 10.1111/codi.70423 (PMC12992198; doi:10.1111/codi.70423)
Supplement: Supplementary file 1 — Supplementary Table 1. Baseline characteristics of 1178 responding patients operated with anterior resection for rectal cancer in Sweden, 2015–2017, by anastomotic leakage. Supplementary Table 2. Baseline characteristics of 600 non‐responding patients operated with anterior resection for rectal cancer in Sweden, 2015–2017, by anastomotic leakage. Supplementary Figure 1. Covariate balance across 10 imputed datasets, for responding patients without permanent stoma. Exposure = leakage versus no leakage. Supplementary Figure 2. Covariate balance across 10 imputed datasets, for responding patients. Exposure = leakage without reintervention versus no leakage. Supplementary Figure 3. Covariate balance across 10 imputed datasets, for responding patients. Exposure = leakage with reintervention versus no leakage. Supplementary Figure 4. Covariate balance across 10 imputed datasets, for all patients. Exposure = leakage vs. no leakage. [file CODI-28-0-s001.docx]

| **Supplementary Table 1.** Baseline characteristics of 1178 responding patients operated with anterior resection for rectal cancer in Sweden 2015–2017, by anastomotic leakage. | | | |
| --- | --- | --- | --- |
|  | No leak (N=1074) | Leak (N=104) | Overall (N=1178) |
| Age |  |  |  |
| Median (IQR) | 67 (60; 72) | 65 (58; 70) | 67 (60; 72) |
| Missing | 1 (0.1%) | 0 | 1 (0.1%) |
| Sex |  |  |  |
| Female | 449 (41.8%) | 37 (35.6%) | 486 (41.3%) |
| Male | 624 (58.1%) | 67 (64.4%) | 691 (58.7%) |
| Missing | 1 (0.1%) | 0 | 1 (0.1%) |
| Body Mass Index (kg/m^2^) |  |  |  |
| Median (IQR) | 25.5 (23.4; 28.2) | 26.1(23.5; 29.0) | 25.6 (23.4; 28.3) |
| Missing | 18 (1.7%) | 0 | 18 (1.5%) |
| ASA |  |  |  |
| I | 279 (26.0%) | 22 (21.2%) | 301 (25.6%) |
| II | 615 (57.3%) | 62 (59.6%) | 677 (57.5%) |
| III-V | 168 (15.6%) | 18 (17.3%) | 186 (15.8%) |
| Missing | 12 (1.1%) | 2 (1.9%) | 14 (1.2%) |
| Hospital volume (annual) |  |  |  |
| Median (IQR) | 20.3 (13.7; 26.3) | 21.7(16.0; 33.3) | 20.3 (14.0; 26.3) |
| Tumour level (cm) |  |  |  |
| ≤ 12 | 805 (75.0%) | 83 (79.8%) | 888 (75.4%) |
| 13-15 | 265 (24.7%) | 19 (18.3%) | 284 (24.1%) |
| Missing | 4 (0.4%) | 2 (1.9%) | 6 (0.5%) |
| Neoadjuvant treatment |  |  |  |
| **Supplementary Table 2.** Baseline characteristics of 600 non-responding patients operated with anterior resection for rectal cancer in Sweden 2015–2017, by anastomotic leakage. | | | |
|  | No leak (N=534) | Leak (N=66) | Overall (N=600) |
| Age |  |  |  |
| Median (IQR) | 67 (60; 74) | 65 (59; 73) | 67 (59; 74) |
| Sex |  |  |  |
| Female | 227 (42.5%) | 23 (34.8%) | 250 (41.7%) |
| Male | 307 (57.5%) | 43 (65.2%) | 350 (58.3%) |
| Body Mass Index (kg/m^2^) |  |  |  |
| Median (IQR) | 26.2 (23.6; 29.1) | 26.0 (22.7; 28.7) | 26.2 (23.4; 29.1) |
| Missing | 5 (0.9%) | 1 (1.5%) | 6 (1.0%) |
| ASA |  |  |  |
| I | 110 (20.6%) | 9 (13.6%) | 119 (19.8%) |
| II | 310 (58.1%) | 38 (57.6%) | 348 (58.0%) |
| III-V | 108 (20.2%) | 17 (25.8%) | 125 (20.8%) |
| Missing | 6 (1.1%) | 2 (3.0%) | 8 (1.3%) |
| Hospital volume (annual) |  |  |  |
| Median (IQR) | 21.0 (14.0; 26.3) | 21.3(14.0; 28.9) | 21.0 (14.0; 26.3) |
| Tumour level (cm) |  |  |  |
| ≤ 12 | 396 (74.2%) | 46 (69.7%) | 442 (73.7%) |
| 13-15 | 137 (25.7%) | 17 (25.8%) | 154 (25.7%) |
| Missing | 1 (0.2%) | 3 (4.5%) | 4 (0.7%) |


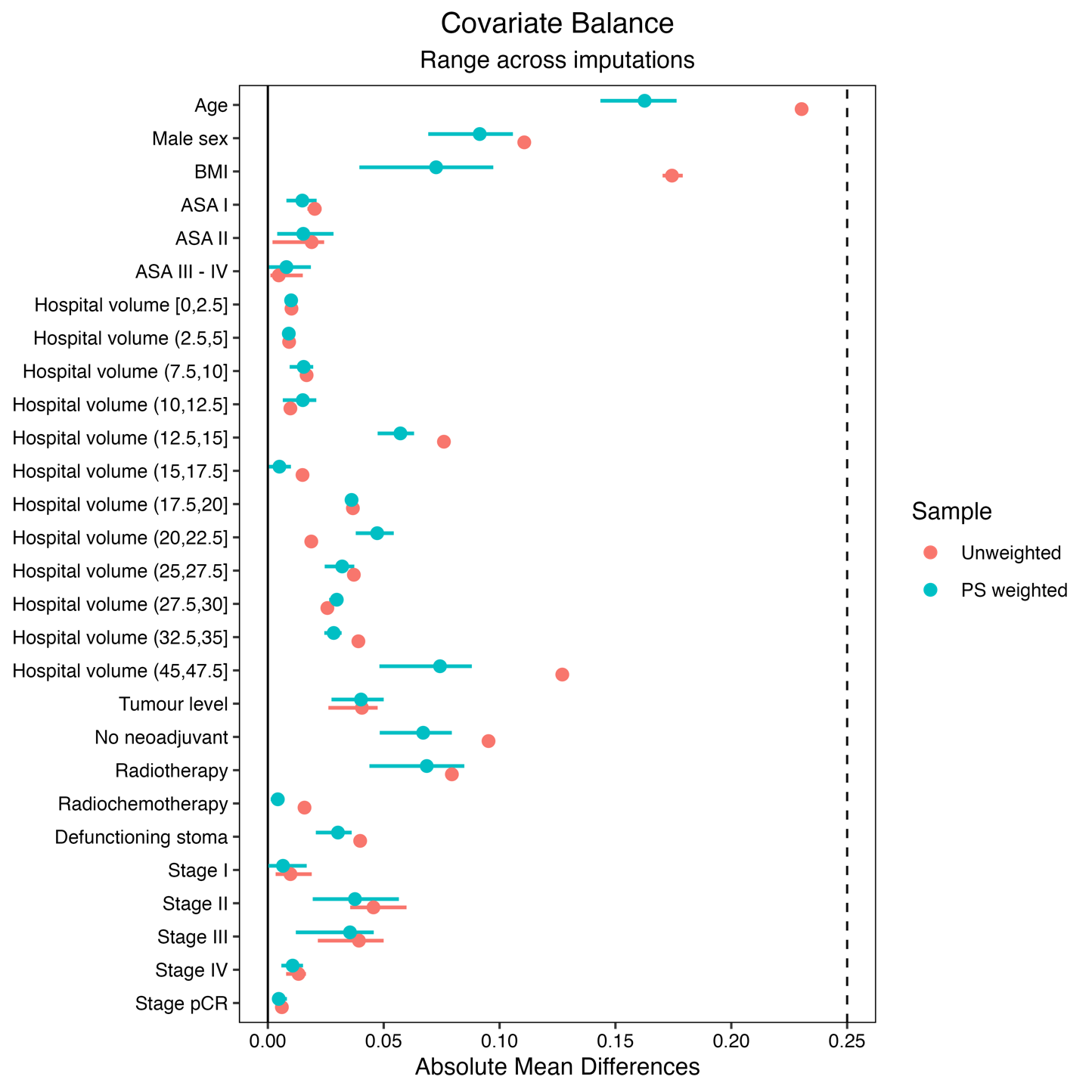


**Supplementary Figure 1.** Covariate balance across 10 imputed datasets, for responding patients without permanent stoma. Exposure = leakage vs. no leakage.


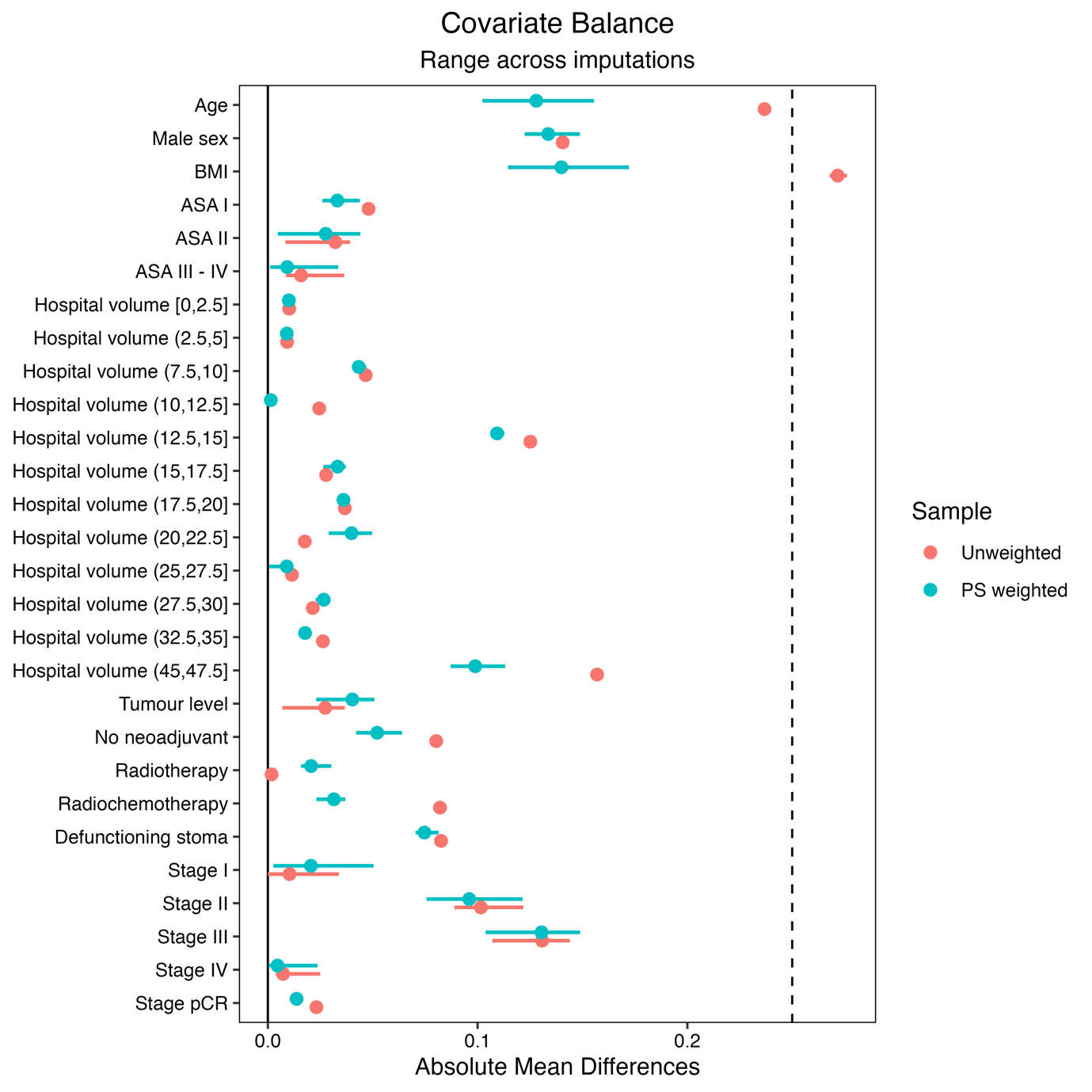


**Supplementary Figure 2.** Covariate balance across 10 imputed datasets, for responding patients. Exposure = leakage without reintervention vs. no leakage.


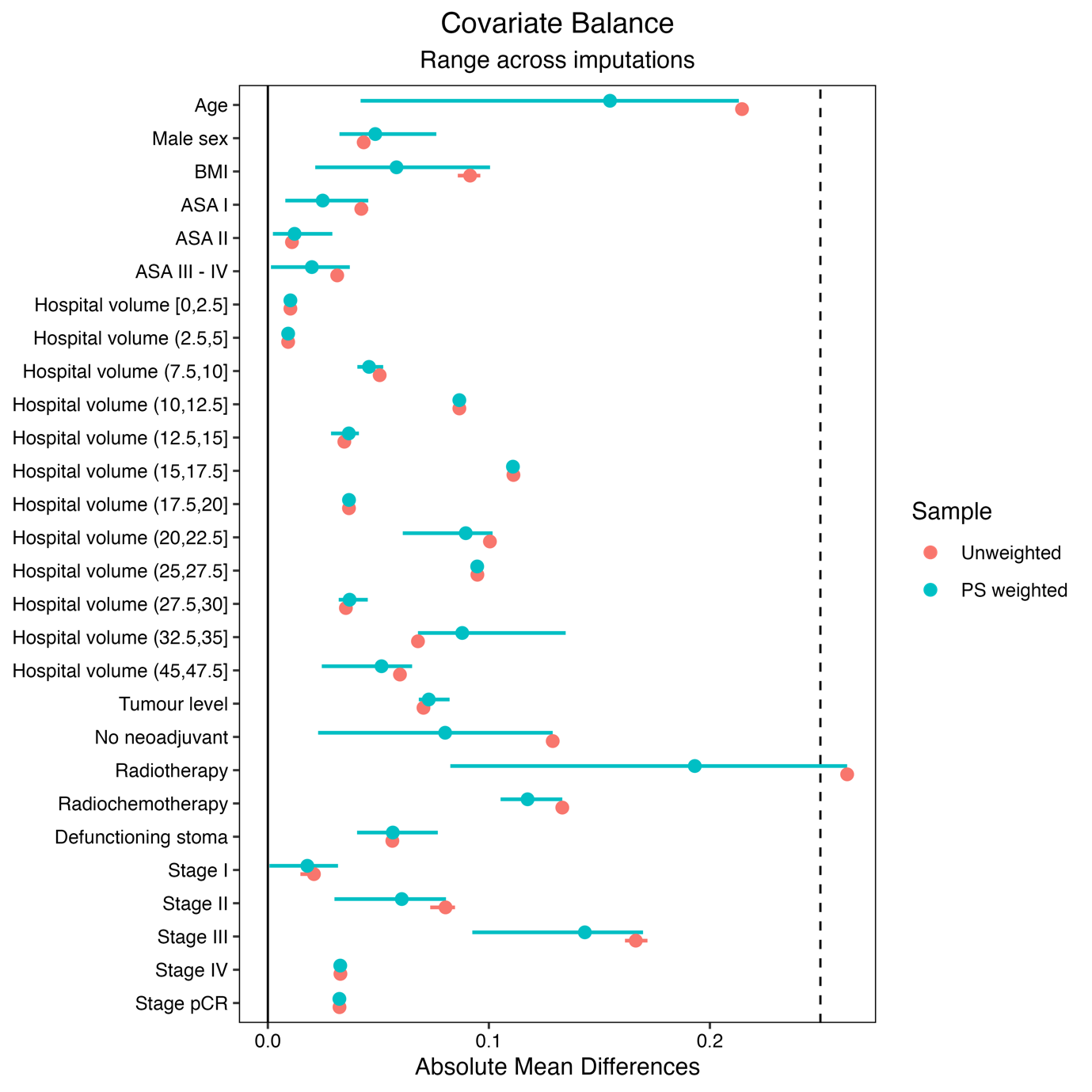


**Supplementary Figure 3.** Covariate balance across 10 imputed datasets, for responding patients. Exposure = leakage with reintervention vs. no leakage.


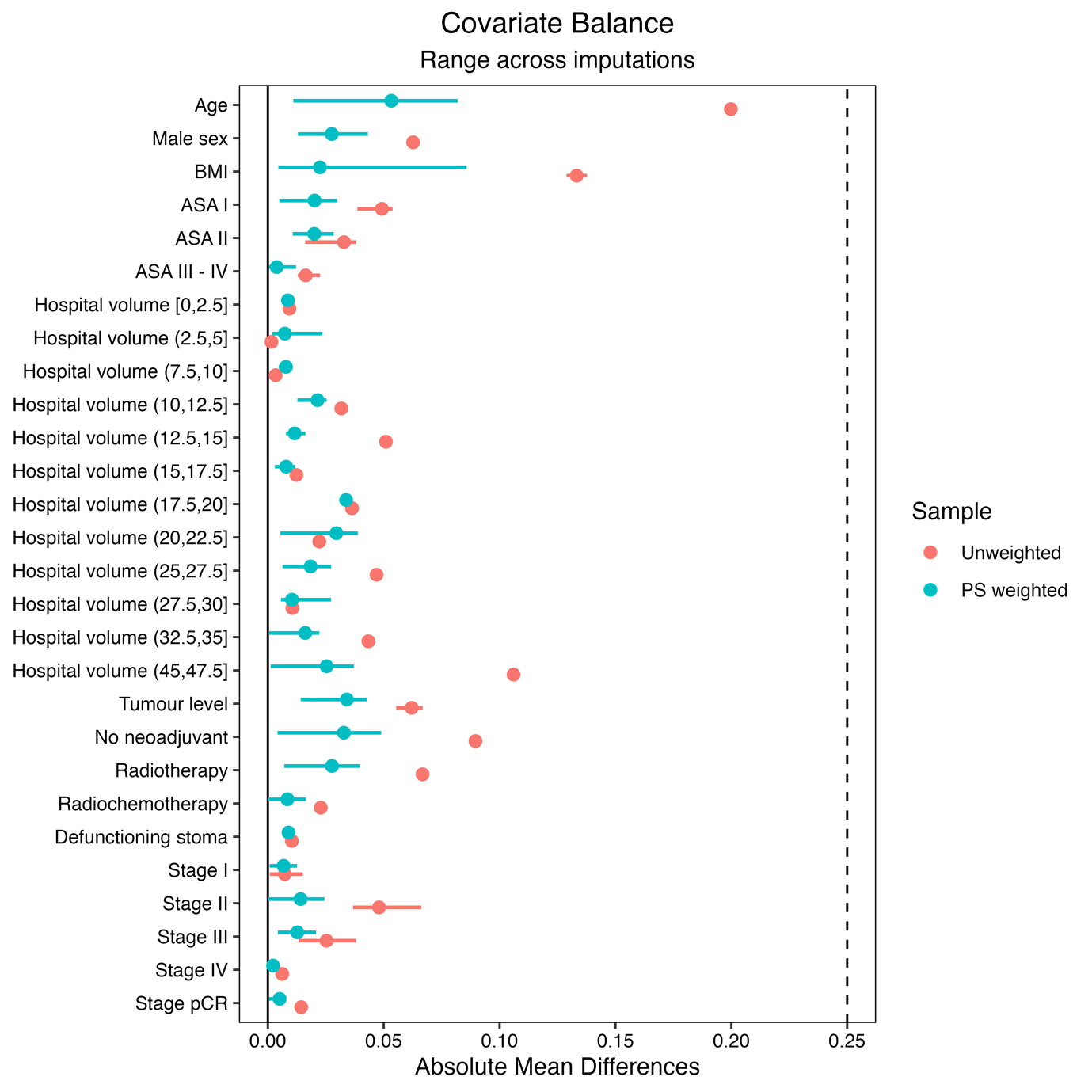


**Supplementary Figure 4.** Covariate balance across 10 imputed datasets, for all patients. Exposure = leakage vs. no leakage.
